# A Review of Telemedicine Guidelines in the South-East Asia Region

**DOI:** 10.1089/tmr.2023.0040

**Published:** 2023-09-25

**Authors:** Parth Sharma, Manik Inder Singh Sethi, Andrian Liem, Hakikat Bir Singh Bhatti, Vatsala Pandey, Anoushka Nair

**Affiliations:** ^1^ Southeast Asia Region, International Working Group for Health System Strengthening, Delhi, India.; ^2^ Department of Community Medicine, Maulana Azad Medical College, Delhi, India.; ^3^ Department of Psychiatry, National Institute of Mental Health and Neurosciences, Bengaluru, Karnataka, India.; ^4^ Public Health Cluster, Jeffrey Cheah School of Medicine and Health Sciences, Monash University Malaysia, Selangor, Malaysia.; ^5^ Department of Computer Science Engineering, Indraprastha Institute of Information Technology, Delhi, India.

**Keywords:** telehealth, telemedicine, policy, e-health

## Abstract

**Introduction:**

Telemedicine use has increased for the past few years, and data security-related issues have also accompanied this. Barriers such as poor digital literacy, unaffordability, and ethical and legal issues have also affected the uptake of digital health. Telemedicine guidelines can help in promoting a suitable environment for wider uptake of telemedicine services by focusing on training, supervision, and monitoring of service providers. This policy review compares the telemedicine guidelines of countries in World Health Organization (WHO) South-East Asia Region (SEAR) as these countries have similar sociocultural backgrounds.

**Methodology:**

Latest telemedicine guidelines of the South Asia Region of the WHO were accessed using the official government websites of the countries. The guidelines that were not in the English language were translated into English using Google Translate. The guidelines were analyzed and presented under the following subheadings: (1) Definitions, Purpose, and Tools of Telemedicine; (2) Clinical Aspects of Telemedicine; and (3) Operational and Technical Aspects of Telemedicine.

**Results:**

Investigating the telemedicine guidelines in the SEAR of the WHO revealed that only 5 out of 11 countries, that is, India, Bangladesh, Thailand, Indonesia and Nepal, have guidelines specifically for telemedicine. Besides Thailand, the other four countries either published (India, Nepal, and Bangladesh) or updated (Indonesia) their telemedicine guidelines after the onset of the COVID-19 pandemic. Guidelines from India and Bangladesh are detailed and robust compared with those from Nepal, Indonesia, and Thailand.

**Conclusion:**

Telemedicine guidelines need to be more robust to improve the uptake of the service. Further research is needed to explore the effectiveness of implementing these guidelines.

## Introduction

Telemedicine, according to the World Health Organization (WHO), is the provision of health care to individuals who are not physically present in the physician's location.^1^ Telemedicine, which was initially used to monitor the health of astronauts, has seen a rapid increase in usage since the COVID-19 pandemic.^2^ In the United States of America, telehealth use increased by 776% in the first 3 months of the pandemic.^3^ An increase in interest and demand for telemedicine was also noticed in 50 countries most affected by the COVID-19 pandemic.^4^

However, a rise in the use of telemedicine was also accompanied by a rise in data breaches.^5^ Cyberattacks have resulted in the interruption of services, economic losses, and even loss of life.^6^ In 2022, a cyberattack at the All-India Institute of Medical Sciences, Delhi, one of the biggest health care institutes in South East Asia, resulted in a loss of 1.4 terabytes of data and an interruption of services for 15 days.^7,8^ Telemedicine is also associated with medical errors such as misdiagnosis, leading to unnecessary prescriptions.^9^

Data safety issues, along with low technical literacy and high costs, have acted as barriers to telemedicine uptake.^10^ Various ethical and legal issues related to telemedicine, such as inappropriate informed consent, patient privacy and confidentiality, data protection, malpractice and professional liability, equity of access, and quality of care, also act as barriers.^11^ It is essential to have a backbone of good governance for a digital ecosystem to thrive. According to WHO, it is essential to combine strategic policy frameworks with oversight, coalition building, regulation, attention to system design and accountability for good governance.^12^ National telemedicine guidelines play a crucial role in addressing existing barriers by facilitating accountability and governance.^13^

Keeping this is mind, the WHO released the “Recommendations on digital interventions for health system strengthening.”^14^ However, it is important to have decentralized guidelines that address the local specific needs of the community. In this review, we compared the latest telemedicine guidelines published by governments of countries belonging to the South-East Asia Region (SEAR) of the WHO, as this region shares socioeconomic and cultural conditions to a great extent.

## Methods

A search for telemedicine guidelines was performed using a sequential approach. At first, a simple Google search was performed using the keywords “Telemedicine Guidelines” along with the country's name. After this, a PubMed search was done to identify published literature on guidelines using the same keywords, and references cited in the published literature were examined to look for the country's latest national guidelines specific for telemedicine. If the guidelines were not found using the first two approaches, the latest guidelines were searched on the websites of the concerned ministries of health of the countries.

The guidelines that were not in the English language were translated into English using Google Translate. Data from the last published telemedicine guidelines were extracted in Microsoft Excel (Microsoft 365) and a comparison between the guidelines of the countries was done. The guidelines were analyzed and presented under the following subheadings: (1) Definitions, Purpose, and Tools of Telemedicine; (2) Clinical Aspects of Telemedicine; and (3) Operational and Technical Aspects of Telemedicine.

## Results

As of May 2023, out of the 11 WHO SEAR countries,^15^ the Maldives, Myanmar, Timor Lester, Bhutan, Democratic People's Republic of Korea, and Sri Lanka did not have any separate national telemedicine guidelines. While Thailand^16^ published its last telemedicine guidelines in 2017, India,^17^ Nepal,^18^ and Bangladesh^19^ published their guidelines in 2020, whereas Indonesia^20^ revised its existing guidelines in 2021, highlighting the impact of the COVID-19 pandemic on telemedicine guidelines.

### Definitions, purpose, and tools of telemedicine

Telemedicine has been defined using various other terms such as “telehealth” (India), “e-health” (Thailand), and “remote health services” (Indonesia). The use of telemedicine in the guidelines ranges from delivering health care services such as diagnosis, treatment, and prevention of disease and injuries to research, patient education, and training of health care providers (Table 1). All guidelines have aimed to improve access to health care services by using telemedicine by addressing the geographical barrier. Telemedicine use was additionally aimed at monitoring patients in self-isolation in Indonesia and Nepal. In India, Nepal, and Bangladesh, the use of telemedicine in case of emergency was allowed if in-person care was not feasible. Indonesia and Thailand mentioned no telemedicine guidelines related to emergency care.

**Table 1.** Purpose and Tools of Telemedicine as Per Country Guidelines

Country	Constituting body	Year of publication	Purpose of telemedicine	Tools for telemedicine
Nepal	Nepal Medical Council, Government of Nepal	2020	Health care provision, patient education, information exchange between health care providers	● Text: Short Message Service, Fax, chat in the platforms such as Facebook Messenger, Viber, and WhatsApp. ● Text with other document, data or image transmission: Chat platforms, e-mail or other internet-based digital systems. ● Audio only: landline telephone, mobile, or cell phone. ● Video recordings and transmission: Stored and forwarded audiovisuals, real-time audio-visual (Skype, Viber, zoom, through other devices connected over LAN, WAN, internet, mobile or chat platforms, etc.) ● Data transferred through imaging or diagnostic devices
India	Medical Council of India in partnership with NITI Aayog, Government of India	2020	Health care provision, patient education, counseling, education of health care providers, research	● Text: Specialized telemedicine smartphone applications, websites, other internet-based systems, and other general messaging/text/chat platforms such as WhatsApp, Google Hangouts, Facebook Messenger, and. E-mail/Fax. ● Video: Telemedicine facility, Apps, Video on chat platforms, Skype/Face time, etc. ● Audio: Telephone or cell phone
Bangladesh	Bangladesh Medical and Dental Council, Government of Bangladesh	2020	Health care provision, patient education, education of health care providers, counseling, information exchange between health care providers	● Text: Specialized telemedicine smartphone applications, websites, other internet-based systems, and other general messaging/text/chat platforms such as WhatsApp, Google Hangouts, Facebook Messenger, and. E-mail/Fax. ● Video: Telemedicine facility, Apps, Video on chat platforms, Skype/Facetime, etc. ● Audio: Telephone or cell phone
Indonesia	Ministry of Health, Government of Indonesia	2021	Health care provision, education of health care providers, information exchange between health care providers	● Tele-radiology, tele-ultrasonography, tele-electrocardiography, clinical teleconsultation, other teleconsultations; telemedicine app to monitor COVID-19 patients who are doing self-quarantine
Thailand	Ministry of Public Health, Government of Thailand	2017	Health care provision except for the sale of drugs, information exchange between health care providers	● Text: In-app chat/messenger. ● Video: Videoconferencing applications such as FaceTime or LINE

LAN, Local Area Network; NITI, National Institution for Transforming India; WAN, Wide Area Network.

Tools of telemedicine have been broadly classified in the guidelines based on the mode of communication: video, audio, and text-based. Except for Indonesia, all countries with guidelines have explicitly mentioned the mode of communication for telemedicine.

### Clinical aspects of telemedicine

All guidelines allow only Registered Medical Practitioners (RMPs) to use telemedicine. The practitioners in Nepal and Indonesia need to be well-versed in the principles and the technology of telemedicine. In India and Bangladesh, RMPs must complete a mandatory online course to provide online consultations. In Thailand, health care providers need to file a form with their health authority to add telemedicine to their list of services. In addition, guidelines from India and Bangladesh have barred technology platforms based on artificial intelligence or machine learning from counseling or prescribing medicines to a patient.

In India, Nepal, and Bangladesh, the RMPs should ensure that a mechanism for the patient to verify the credentials and contact details of the RMP exists. However, the guidelines have not mentioned the mechanism to be followed for the process of verification. In the guidelines for Thailand and Indonesia, specific instructions for patient verification are not mentioned. However, the RMPs are expected to follow the country's standard operating procedures and regulations related to health services. The RMPs in India, Nepal, and Bangladesh must verify and confirm the patient's identity by name, age, gender, address, and phone number. For minors, teleconsultation can be done in India, Nepal, and Bangladesh if the minor consults with an adult after their identity and relationship with the patient are ascertained. Such provisions are not mentioned in the guidelines of Thailand and Indonesia.

Cross-border teleconsultation can be practiced in Nepal only in the presence of a local RMP, with the ethical and legal liabilities being borne by the practitioner in Nepal. In Thailand, cross-border teleconsultations can be done with countries with adequate data protection standards, per Thailand's Data Protection Committee. The guidelines from India, Bangladesh, and Indonesia do not mention cross-country telemedicine provisions.

The guidelines from India, Nepal, and Bangladesh have strictly mentioned acts that are not permissible while providing teleconsultations, as opposed to Indonesia and Thailand, which have not outlined such acts in their guidelines (Table 2). Guidelines for prescribing drugs are restrictive in India, Nepal, Bangladesh, and Indonesia, whereas prescribing drugs over teleconsultation is not permitted in Thailand (Table 2).

**Table 2.** Guidelines for Prescription Drugs and Code of Conduct of Health Care Provider

Country	Drugs that can be prescribed using telemedicine	Other restrictions
Nepal	Prescribing medications through telemedicine consultation is at the professional discretion of the RMP. Drugs listed under the category “A” under drug category rules 2043 (1986)—narcotic and poisonous drugs—cannot be prescribed using telemedicine.	● RMP should ensure the privacy of the patient or client while providing telemedicine services. The presence of other people during the communication should be made aware to the patient or client. ● Any professional code of conduct breach will be subject to disciplinary actions as per the Nepal Medical Council Act 1964 and bylaws
India	Prescribing medications through telemedicine consultation is at the professional discretion of the RMP. The categories of medicines that can be prescribed are listed below: List O: Medications that are safe to be prescribed through any mode of teleconsultation. List A: Medications can be prescribed during the first consult, which is a video consultation, and are being re-prescribed for a refill in case of follow-up. List B: Medications that RMP can prescribe to a patient who is undergoing a follow-up consultation in addition to those that have been defined during an in-person consult for the same medical condition. Prohibited List: An RMP providing consultation through telemedicine cannot prescribe medicines on this list as they have a high potential for abuse and could harm the patient or society if misused.	RMPs cannot: ● Insist on telemedicine when the patient is willing to travel to a facility and requests an in-person consultation. ● Misuse patient images and data, especially private and sensitive. ● Prescribe medicines from the specific restricted list. ● Solicit patients for telemedicine through any advertisements or inducements.
Bangladesh	Similar as India's guidelines	Similar as India's guidelines
Indonesia	Drug prescriptions can be given electronically, except for drug prescriptions for psychotropic substances.	The RMPs should not provide telemedicine services if the provided data cannot be appropriately interpreted.
Thailand	RMP is not permitted to prescribe drugs using telemedicine.	● Being informed about the details of their treatment under the telemedicine service ● RMPs should inform patients that not all diseases or medical conditions can be treated through telemedicine services. ● RMPs should assure patients that the telemedicine service complies with relevant laws governing medical devices and pharmaceutical practices when using AI.

AI, artificial intelligence; RMPs, Registered Medical Practitioners

### Operational and technical aspects of telemedicine

Obtaining consent from the patient is necessary for all five countries with telemedicine guidelines. It has to be explicit in Thailand, Indonesia, and Bangladesh, whereas in India and Nepal, the consent could be implied or detailed depending on the scenario. Except for Nepal, the guidelines make it necessary for RMPs to maintain records of the telemedicine services provided. However, how these records should be maintained and for what duration has not been mentioned in any guidelines.

Except for Nepal, guidelines for telemedicine platforms have been mentioned by each country. These guidelines are stricter for India and Bangladesh, where the technology platforms providing telemedicine services must ensure that the RMPs are registered with the respective national medical councils, their details are provided on the forum, and a grievance redressal mechanism is in place before initiating services.

Telemedicine platforms in Thailand and Indonesia must follow the countries' existing data safety and security laws of the countries, that is, Thailand's Personal Data Protection Law (2022) and the Personal Data Protection Law of Indonesia (2022), respectively. Besides mentioning existing laws, guidelines from India, Nepal, and Bangladesh mention that it is the responsibility of the RMP to maintain confidentiality and data safety. However, as per these guidelines, if confidentiality is compromised due to the technology platform, the RMP will not be held liable. Other laws in SEAR countries governing data safety and privacy have been summarized in Table 3.

**Table 3.** Laws Governing Data Privacy in South-East Asia Region Countries

Country^*^	Laws governing data privacy and safety	Key highlights
Nepal	Individual Privacy Act (2018) and Individual Privacy Regulation (2020)	These acts list cover protection of sensitive information such as caste, ethnicity, political affiliation, religious faith, physical or mental health, and sexual orientation. Data collection is permitted only with the consent of the person concerned, and the acts mandate that the collected data should be used only for the purpose for which it was collected. The acts also have provisions for processing sensitive information in the context of public health protection, disease identification, and health treatment.
India	India's IT Act (2000), the SPDI Rules (2011), the draft National Data Governance Framework Policy, DISHA (2017), the Ayushman Bharat Digital Mission, and the draft Health Data Management Policy by National Health Authority	These policies and acts focus on the obligations of data fiduciaries about the processing of personal data. They require transparency, privacy by design, purpose limitation, collection and use restrictions, and principal data empowerment. DISHA specifically dictates that any data transfer of digital health data by a clinical establishment or entity can only occur upon the receipt of the consent of the owner, who has been informed of their rights under DISHA and is aware of the purposes of collecting their digital health data.
Bangladesh	Information and Communication Technology Act (2006), Digital Security Act (2018)	The Information and Communication Technology Act provides legal recognition for electronic certificates and transactions, including implementing and maintaining reasonable security practices for handling sensitive personal data. Health data are not expressly defined. The Digital Security Act aims to ensure national data security and includes data crime prevention and trial provisions. If any data published threaten data security, the Director General can request the relevant authority to remove or block the data. No specific law exists for health data protection, but a draft digital strategy is under consideration, which includes objectives for ensuring the privacy and security of personal health information.
Indonesia	PDPA (2022), MOH Regulation No. 24 of 2022	The PDPA sets definitions and principles for personal data handling, including consent, data updating, breaches, transfers, and sanctions. The MOH Regulation No. 24 of 2022 specifically governs personal health data, outlining obligations for storage, deletion, and confidentiality of medical records, encompassing a patient's identity, examinations, medications, and related services. Both define health data as records or information about an individual's physical and mental health or health services.
Thailand	Thailand Personal Data Protection Act (2019)	Under the Thailand PDPA, explicit consent is needed to collect sensitive personal data, with certain exemptions. It is allowed when mitigating danger to the data subject's life or health or when it's necessary for legal claims or compliance. This can include preventive medicine, employee health assessment, medical diagnosis, care system management, public health interests, employment protection, social security, research purposes, and substantial public interests. Data can also be collected by authorized bodies or nonprofit organizations for their legitimate activities.
Sri Lanka	Personal Data Protection Act of Sri Lanka (No. 9 of 2022)	The PDPA of Sri Lanka (No. 9 of 2022) outlines key points for a data privacy policy. It defines “health data” as personal data related to the physical or psychological health of an individual. Lawful processing is allowed for public health purposes, including the management of health care services and the control of communicable diseases. Processing is also permitted for employment, social security, public safety, and emergencies threatening life, health, or safety. Personal data made public, legal claims, and public interest determined under Schedule I are covered. Processing by licensed health professionals for medical purposes is allowed. Suitable measures must safeguard the data subject's rights.
Myanmar	Law Protecting the Privacy and Security of Citizens, 2017 (as amended in 2020 and 2021)	Myanmar lacks a specific data privacy law, but existing legislation includes provisions for privacy protection. The Constitution recognizes citizens' privacy and security rights. The Electronic Transactions Law, amended in 2021, covers personal data protection. The Law for Protection of Privacy and Security of Citizens, although suspended, addresses personal privacy and security. A draft cybersecurity law aims to enhance data privacy. Government agencies have interception powers, and limitations exist on data subject rights. Cross-border data transfers require consent, and the Post and Telecommunications Department oversees data transfer control.
Bhutan	Guidelines on Data Privacy and Data Protection 2022	These Bhutanese guidelines, titled “Guidelines on Data Privacy and Data Protection 2022,” govern data privacy and safety for FSPs. They particularly emphasize the protection of sensitive personal data or information, which includes passwords, location data, IP addresses, financial details, physical, physiological, and mental health conditions, medical records, and biometric information. FSPs are obligated to uphold “Privacy by Design and Default,” systematically mitigate privacy risks, and align with international standards. This accountability aims to foster public trust in the financial and economic data sectors while considering the implications of digitization in Bhutan.

^*^ *Note:* Specific data privacy laws for Timor-Leste, Maldives, and the Democratic People's Republic of Korea (North Korea) were not publicly available through open internet sources at the time of this study.

DISHA, Digital Information Security in Healthcare Act; FSPs, financial service providers; MOH, Ministry of Health; PDPA, Personal Data Protection Act.

The cost of teleconsultations can be decided by the RMPs as per the guidelines of India, Bangladesh, and Nepal. However, the guidelines from Nepal recommend that the fees for teleservices not exceed those for traditional in-person care. The telemedicine costs in Indonesia are covered by the government with a reimbursement system where health service providers must make claims for the provided telemedicine services.

The summary of the comparison of telemedicine guidelines in SEAR countries is highlighted in Table 4.

**Table 4.** Comparison of Telemedicine Guidelines in South-East Asia Region

Country	Definition and purpose of telemedicine	Tools for telemedicine	Specific guidelines											
			Emergency care	Information exchange	Identification of HCW	Identification of patient	Data safety	Cross border telemedicine	Cost of service	Consent	Drug prescription	Technology platforms	Documentation	Training in telemedicine
Nepal	✓	✓	✓	✓	✓	✓	✓	✓	✓	Implied/explicit	✓	—	—	—
India	✓	✓	✓	✓	✓	✓	✓	—	✓	Implied/explicit	✓	✓	✓	✓
Bangladesh	✓	✓	✓	✓	✓	✓	✓	—	✓	Explicit	✓	✓	✓	✓
Indonesia	✓	✓	—	✓	✓	—	✓	—	✓	Explicit	✓	✓	✓	—
Thailand	✓	✓	—	✓	✓	—	✓	✓	—	Explicit	Not permitted	✓	✓	—

✓, mentioned in the guidelines.

HCW, health care worker.

## Discussion

The COVID-19 pandemic has demonstrated the potential of telemedicine in overcoming barriers to health care access and delivery, particularly in regions with limited resources and vast populations, such as the WHO SEAR. With a collective population of more than 2 billion people and significant disparities in health care infrastructure and services, this region faces unique challenges and opportunities in the adoption and sustainability.

The use of telehealth services had been on a steady increase globally for the past decade, but the overall growth had remained slow. The COVID pandemic catalyzed the uptake of telemedicine and its use grew multifold. The western world was the first to spring into action as there was some provision of telehealth already in place. The ease of adoption of these services was further facilitated by laws passed by their national governments for use of telehealth.^21^

Most of the countries in SEAR are using telehealth services for health care provision, education of health care providers, and information exchange between health care providers and patients. Most of these countries have guidelines in place for prescription of medicines, with the exception of Thailand, which does not allow medicines to be prescribed through a teleconsultation, and Indonesia, where telemedicine can only be conducted at registered health facilities. Although with certain restrictions such as narcotic drugs and drugs that may have a higher potential for misuse. This contrasts with United States where in a lot of states permit dispensing opioid medication through telemedicine.^22^

A well-trained health care workforce plays a central role in the successful implementation of telemedicine.^23^ Our review identifies varying levels of human resource capacity and training programs across the region. Shortages of skilled telemedicine professionals, limited digital literacy among health care providers, and the need for specialized training in telemedicine technologies are common challenges.^10,24,25^ To ensure sustainability, comprehensive capacity-building initiatives, including training programs, continuing education, and the establishment of telemedicine-specific curricula, should be prioritized in the region's health care systems.

The financial sustainability of telemedicine services is a critical aspect that demands attention in policy formulation. Our analysis indicates that although some countries in the WHO SEAR have implemented reimbursement mechanisms for telemedicine services, others lack sustainable funding models. It is known that government-funded insurance schemes improve access to care and provide financial risk protection to individuals.^26^ Therefore, including telemedicine as a part of these the government-funded insurance could possibly improve its utilization.

Data privacy is a critical aspect of telemedicine, and several problems can arise concerning the protection of patient data. Over the years we have seen a lot of data breaches.^27^ Telemedicine involves the transmission and storage of patient data over digital platforms, making it susceptible to data breaches. Unauthorized access, hacking, or technical vulnerabilities can compromise patient privacy and confidentiality. Implementing robust security measures, such as encryption and secure data storage systems, is essential to prevent data breaches. Unfortunately, most of the countries of this region do not have robust data protection laws in place.

Although the authors have done their best to be robust with the methodology, but the fact that some of the information was in the local language or not in public domain, there is a possibility that some of the information could have been overlooked. There is a need for a more robust policy review with the stakeholders of the respective countries and further research may be undertaken to check for the effectiveness of implementation of the telemedicine guidelines in these countries.

We hope that this policy review on telemedicine in the SEAR has implications in the form of development or enhancement of regulations and governance frameworks specifically tailored to telemedicine. We believe that telemedicine has the potential to address health care access challenges, especially in remote or underserved areas. By conducting this policy review, we sincerely hope this information can inform policy decisions and resource allocation strategies to maximize the benefits of telemedicine while ensuring affordability and financial viability.

Policymakers should consider incorporating cross-border teleconsultation provisions, strengthening data privacy regulations, and developing guidelines for emerging technologies such as artificial intelligence. Collaboration among countries in the SEAR can facilitate knowledge sharing, capacity building, and the development of best practices to drive the sustainable and equitable adoption of telemedicine.

## Conclusion

In conclusion, the policy review on telemedicine in the SEAR has revealed significant variations in the adoption and implementation of telemedicine guidelines among the 11 WHO SEAR countries. Although some countries have established comprehensive guidelines, others have yet to develop national telemedicine policies. The guidelines uniformly emphasize the goal of improving health care access by addressing geographical barriers and facilitating remote consultations. They recognize the importance of technology platforms and classify telemedicine tools based on communication modes. The guidelines also underscore the need for RMPs to ensure quality and safety in teleconsultations, with requirements ranging from completion of online courses to adherence to country-specific operational procedures.

## Authorship Contribution Statement

Conceptualization, methodology, data curation, and writing and reviewing final draft by P.S. Data curation and writing original draft by M.I.S.S. Data curation and reviewing final draft by A.L. Data curation by H.B.S.B., V.P., and A.N.

## Abbreviations Used

AI: Artificial Intelligence
DISHA: Digital Information Security in Healthcare Act
FSPs: financial service providers
MOH: Ministry of Health
PDPA: Personal Data Protection Act
RMPs: Registered Medical Practitioners
SEAR: South-East Asia Region
WHO: World Health Organization
